# Coronary artery bypass grafting with or without preoperative physiological stenosis assessment: a SWEDEHEART study

**DOI:** 10.1093/eurheartj/ehaf327

**Published:** 2025-05-16

**Authors:** Emma C Hansson, Elmir Omerovic, Dimitrios Venetsanos, Joakim Alfredsson, Andreas Martinsson, Björn Redfors, Amar Taha, Susanne J Nielsen, Anders Jeppsson

**Affiliations:** Department of Cardiothoracic Surgery, Sahlgrenska University Hospital, Blå Stråket 5, plan 5, Gothenburg S-413 45, Sweden; Department of Molecular and Clinical Medicine, Institute of Medicine, Sahlgrenska Academy, University of Gothenburg, SU Sahlgrenska, Gothenburg S-413 45, Sweden; Department of Molecular and Clinical Medicine, Institute of Medicine, Sahlgrenska Academy, University of Gothenburg, SU Sahlgrenska, Gothenburg S-413 45, Sweden; Department of Cardiology, Sahlgrenska University Hospital, Blå Stråket 3, Gothenburg S-413 45, Sweden; Division of Cardiology, Department of Medicine, Karolinska Institute Solna and Karolinska University Hospital, Stockholm S-17177, Sweden; Department of Health, Medicine and Caring Sciences, Linköping University, Linköping S-58183, Sweden; Department of Cardiology, Linköping University, Linköping S-58183, Sweden; Department of Molecular and Clinical Medicine, Institute of Medicine, Sahlgrenska Academy, University of Gothenburg, SU Sahlgrenska, Gothenburg S-413 45, Sweden; Department of Cardiology, Sahlgrenska University Hospital, Blå Stråket 3, Gothenburg S-413 45, Sweden; Department of Molecular and Clinical Medicine, Institute of Medicine, Sahlgrenska Academy, University of Gothenburg, SU Sahlgrenska, Gothenburg S-413 45, Sweden; Department of Cardiology, Sahlgrenska University Hospital, Blå Stråket 3, Gothenburg S-413 45, Sweden; Department of Molecular and Clinical Medicine, Institute of Medicine, Sahlgrenska Academy, University of Gothenburg, SU Sahlgrenska, Gothenburg S-413 45, Sweden; Department of Cardiology, Sahlgrenska University Hospital, Blå Stråket 3, Gothenburg S-413 45, Sweden; Department of Cardiothoracic Surgery, Sahlgrenska University Hospital, Blå Stråket 5, plan 5, Gothenburg S-413 45, Sweden; Department of Molecular and Clinical Medicine, Institute of Medicine, Sahlgrenska Academy, University of Gothenburg, SU Sahlgrenska, Gothenburg S-413 45, Sweden; Department of Cardiothoracic Surgery, Sahlgrenska University Hospital, Blå Stråket 5, plan 5, Gothenburg S-413 45, Sweden; Department of Molecular and Clinical Medicine, Institute of Medicine, Sahlgrenska Academy, University of Gothenburg, SU Sahlgrenska, Gothenburg S-413 45, Sweden

**Keywords:** Coronary artery bypass graft, Fractional flow reserve, Instantaneous wave-free ratio

## Abstract

**Background and Aims:**

Physiological flow assessment of coronary stenoses, such as fractional flow reserve, are routinely used to guide percutaneous coronary intervention, but it has not been equally recognized to guide coronary artery bypass grafting (CABG). Mid-term outcomes in CABG patients with and without preoperative flow assessment were compared.

**Methods:**

All patients with first-time isolated CABG in Sweden 2013-2020 were identified in the SWEDEHEART registry (*n* = 18 211), which also provided information on flow assessment. Data were linked with three mandatory national registries. Median follow-up was 3.6 years (range 0–7.5). Incidence of all-cause mortality, stroke, new myocardial infarction, new coronary angiography, and new revascularization was compared using adjusted Cox regression models. The proportional hazard assumption was violated for new angiography and revascularization. Hence, follow-up was divided into 0–2 and >2 years.

**Results:**

Overall, 2869 patients (15.8%) had flow assessment before surgery, increasing from 7.1% in 2013% to 21.5% in 2020. Patients with flow assessment were younger, had a lower EuroSCORE II, and received fewer distal anastomoses (3.0 ± 0.9 vs 3.2 ± 1, *P* < .001). There were no associations between flow assessment and mortality, post-discharge myocardial infarction, or stroke. New angiography and new revascularization were not significantly different 0–2 years, but preoperative flow assessment was associated with a higher risk for new angiography [adjusted hazard ratio (aHR) 1.32, 95% confidence interval (CI) 1.08–1.62, *P* = .008] and new revascularization (aHR 1.55, 95% CI 1.18–2.04, *P* = .002) >2 years after CABG.

**Conclusions:**

Preoperative flow assessment was not associated with improved clinical outcomes but with a higher risk for new angiography and new revascularization >2 years after CABG. The results suggest that the use of flow assessment with current cut-off levels may not be applicable in CABG, and further studies are needed.

## Structured Graphical Abstract

**Structured Graphical Abstract:**
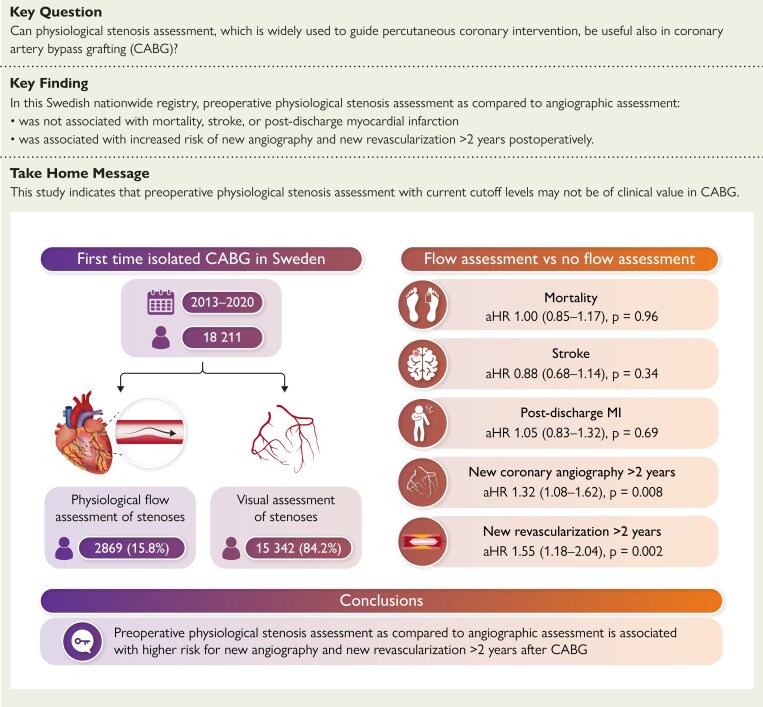

**See the editorial comment for this article ‘Physiological stenosis assessment for coronary artery surgery: quo vadis?’, by P. Kolh *et al*., https://doi.org/10.1093/eurheartj/ehaf318.**

## Introduction

Coronary artery bypass grafting (CABG) maintains a Class I indication in current international guidelines for treating multivessel coronary artery disease,^1,2^ recommending complete revascularization of all vessels with significant stenoses. The degree of stenosis is traditionally visually determined from a coronary angiogram. Still, for percutaneous coronary intervention (PCI), the same guidelines strongly recommend using physiological flow assessment such as fractional flow reserve (FFR) or instantaneous wave-free ratio (IFR) to determine significance in intermediate or uncertain stenoses, especially in stable coronary syndrome patients.^1,2^ These methods and other similar methods with slight procedural variations have been shown to improve short- and long-term outcomes after PCI in several studies.^3-6^ In patients undergoing CABG, studies of preoperative flow assessment have resulted in conflicting evidence, where smaller randomized studies with short follow-up indicate that flow assessment guidance may predict improved graft patency after CABG, especially in arterial grafts, but without clear benefit on clinical outcomes.^7-13^ Consequently, guidelines have not endorsed using flow assessment to guide clinical decision-making in CABG patients but instead called for large-scale, preferably randomized, studies.^1^ Large population-based observational studies comparing mid-term or long-term outcomes in CABG patients, evaluating the use of flow assessment as part of the preoperative work-up, are also lacking.

Therefore, this study aimed to describe the use of flow assessment before CABG over time in a large nationwide cohort study and compare short- and mid-term outcomes after CABG between patients with and without flow assessment prior to surgery.

## Methods

### Patients and data sources

**Figure 1:**
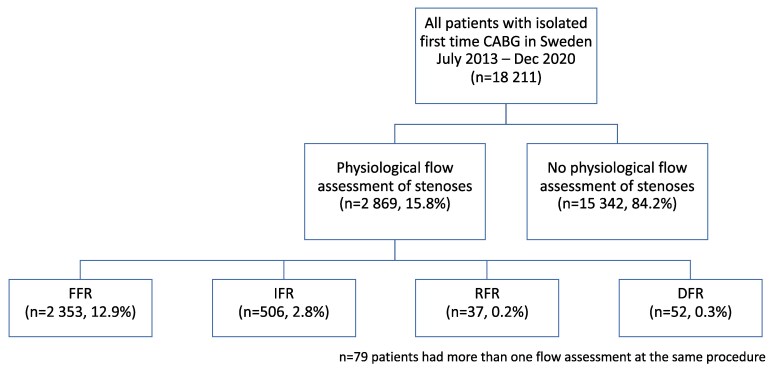
Flowchart of patients included in the study (CABG, coronary artery bypass grafting; FFR fractional flow reserve; IFR, instantaneous wave-free ratio; RFR, resting full-cycle ratio; DFR, diastolic hyperaemia-free ratio)

This observational nationwide cohort study included all 18 211 patients who underwent first-time isolated CABG in Sweden from 2013 to 2020 (*Figure 1*). Data were collected from the SWEDEHEART (Swedish Web-system for enhancement and development of evidence-based care in heart disease evaluated according to recommended therapies) registry,^14^ which is a combination of subspecialized national quality registries dedicated to different aspects of cardiac care in Sweden, e.g. cardiac surgery and PCI. Patients were identified in the Swedish Cardiac Surgery Registry,^15^ which is a part of SWEDEHEART. This registry also supplied data on surgical variables such as graft selection, operation times, and complications during the index hospitalization. Data from the Cardiac Surgery Registry were merged with information on flow assessment used during the preoperative angiography from the Swedish Coronary Angiography and Angioplasty Registry.^14^ Subsequently, data were linked with the National Patient Register and the National Cause of Death Register using the unique identifying personal number given to every Swedish citizen at birth or immigration. As these are mandatory registries, only patients who emigrated during the study period (*n* = 65, 0.4%) were lost to follow-up and were censored at the time of emigration. The National Patient Register contains information on all International Classification of Diseases versions 9 and 10 (ICD-9 and ICD-10) diagnoses from every hospitalization in Sweden since 1987 with complete coverage and excellent validity.^16^ This registry supplied information on comorbidity, new hospitalizations and new diagnoses during the follow-up. Since its establishment in 1952, the National Cause of Death Register has recorded the date and cause of all deaths in Sweden.^17^

The study was approved by the Swedish Ethical Review Authority (registration number 2021-00122, approved 31 March 2021). The study was performed in accordance with the Declaration of Helsinki and the present manuscript was written in accordance with the Strengthening the Reporting of Observational Studies in Epidemiology recommendations.^18^

### Preoperative assessment and surgical procedures

Flow assessment was used at the discretion of the interventional cardiologist performing the coronary angiography. In some patients two different flow assessment methods were used, either because the patient was included in a clinical trial comparing flow assessment methods or at the discretion of the interventionist. Flow assessment data are generally presented at Heart Team discussions of the individual patients but are optional as guidance for surgical revascularization as per current recommendations in guidelines.^1^ Surgical considerations such as on- or off-pump and choice of grafts (arterial or venous) are not standardized in Sweden but are left to the surgeon’s discretion.

### Outcomes

The early endpoint was 30-day all-cause mortality. Mid-term endpoints were time to all-cause death, and time to first new hospitalization due to myocardial infarction (MI) after discharge from index hospitalization, first post-discharge stroke, first new coronary angiography, and first new revascularization, including both PCI and CABG. Information on these endpoints was collected from the National Patient Register.^16^ The ICD codes used are listed in Supplementary data online, *Table S1*.

### Statistics

Comparisons between groups at baseline were performed using the Wilcoxon rank sum test for continuous variables and Fisher’s exact test for categorical variables and presented as frequency with percent, mean with standard deviation, or median with 25th −75th percentiles (interquartile range, IQR). The inverse Kaplan–Meier method was used to calculate median follow-up time. Early and mid-term endpoints were compared between groups using Cox regression models adjusted for the indication for angiographic evaluation (acute coronary syndrome/chronic coronary artery disease), age, sex, body mass index, estimated glomerular filtration rate, previous PCI, left ventricular ejection fraction (LVEF), priority (acute/non-acute), New York Heart Association class, EuroSCORE II, previous MI, diabetes mellitus, hypertension, heart failure, atrial fibrillation, previous stroke, chronic respiratory disease, renal failure, peripheral vascular disease, history of cancer, hyperlipidaemia, and year of surgery. There were missing values in the dataset used for the regression models in the variables body mass index, creatinine clearance, LVEF, and previous PCI (*Table 1*). Cardiopulmonary bypass (CPB) time was excluded from the regression analysis due to the high proportion of missing data. None of the variables exceeded the 5% threshold, ensuring that imputation could be performed effectively without introducing significant bias, and they were imputed using the missRanger package in R, which utilizes random forest imputation to fill in missing values.^19^ This imputation method employs a random forest imputation technique that replaces the missing data with estimates. The missRanger method is highly accurate in imputing missing data points while still maintaining the distribution of the data.^19^ The proportion of missing values regarding operation times and grafting details, not used in the regression models, are reported in *Table 2*.

**Table 1:** Baseline variables in patients with and without preoperative flow assessment

	Flow assessment (*n* = 2869)	No flow assessment (*n* = 15 342)	*P*-value
Age (years)	68 (61–73)	70 (63–75)	<.001
Female sex	426 (14.8%)	2795 (18.2%)	<.001
BMI (kg/m^2^) Unknown	27.5 (25.2–30.4) 60	27.2 (24.7–30.0) 627	<.001
Diabetes	975 (34.0%)	4847 (31.6%)	.012
Hypertension	2024 (70.5%)	10 384 (67.7%)	.003
Heart failure	373 (13.0%)	1983 (12.9%)	>.9
Atrial fibrillation	315 (11.0%)	1910 (12.4%)	.027
Previous stroke	159 (5.5%)	1067 (7.0%)	.005
History of cancer	434 (15.1%)	2596 (16.9%)	.018
Chronic respiratory disease	309 (10.8%)	1499 (9.8%)	.10
Peripheral vascular disease	254 (8.9%)	1365 (8.9%)	>.9
Previous PCI Unknown	851 (29.7%) 1	2826 (17.1%) 12	<.001
Acute coronary syndrome	1575 (54.9%)	10 432 (68.0%)	<.001
Ejection fraction			<.001
>50%	2111 (73.6%)	10 457(68.2%)	
31–50%	657 (22.9%)	4002 (26.1%)	
21–30%	78 (2.7%)	708 (4.6%)	
≤20%	22 (0.8%)	166 (1.1%)	
Unknown	1	9	
Renal failure	139 (4.8%)	763 (5.0%)	.8
eGFR (mL/min) Unknown	89 (71–109) 64	84 (67–105) 631	<.001
EuroSCORE II	1.28 (0.89–1.99)	1.52 (1.01–2.52)	<.001

Median and interquartile range or number and percentage.

BMI, body mass index; eGFR, estimated glomerular filtration rate; PCI, percutaneous coronary intervention.

**Table 2:** Surgical characteristics in patients with and without preoperative flow assessment

	Flow assessment (*n* = 2869)	No flow assessment (*n* = 15 342)	*P*-value
Use of CPB	2836 (98.8%)	15 194 (99.0%)	.36
Median CPB time (min)	73 (57–93) (*n* = 1982)	76 (60–96) (*n* = 8368)	<.001
Median aortic cross-clamp time (min)	46 (36–60) (*n* = 1982)	48 (38–62) (*n* = 8368)	<.001
Distal anastomoses			
Mean and SD	2.95 ± 0.92	3.20 ± 0.96	<.001
Median and IQR	3 (2–3) (*n* = 2624)	3 (3–4) (*n* = 12 653)	<.001
Use of left internal mammary artery	2511 (95.7%) (*n* = 2624)	12 045 (95.2%) (*n* = 12 653)	.27
Bilateral internal mammary artery	291 (11.1%) (*n* = 2624)	595 (4.7%) (*n* = 12 653)	<.001
Use of venous grafts	2328 (88.7%) (*n* = 2624)	11 864 (93.7%) (*n* = 12 653)	<.001
Use of radial artery	38 (1.4%) (*n* = 2624)	240 (1.9%) (*n* = 12 653)	.13

Data are presented as mean and standard deviation, median and interquartile range, or number (%).

CPB, cardiopulmonary bypass; IQR, interquartile range; SD, standard deviation.

We performed a causal mediation analysis to assess whether the relationship between FFR measurement and repeat revascularization is mediated by the number of anastomoses. The analysis was conducted using the mediation package in R.^20^ For further details on the mediation analysis, see Methods section in the Supplement. Furthermore, we calculated *E*-values to quantify the amount of residual confounding necessary to negate a significant difference in the risk estimates.^21^ The proportional hazard assumption was tested using Schoenfeld residuals according to Grambsch and Therneau.^22^ The proportional hazard assumption was violated for new revascularization and new angiography (treatment-time interaction *P* < .001) but not for death, stroke, or post-discharge MI. Landmark analyses were therefore performed for new revascularization and new angiography, dividing the follow-up into 0–2 years and >2 years, after which the proportional hazards assumption was met. Adjusted hazard ratios (aHR) are presented with 95% confidence intervals (CI). *P*-values <.05 were considered statistically significant.

## Results

### General

**Figure 2:**
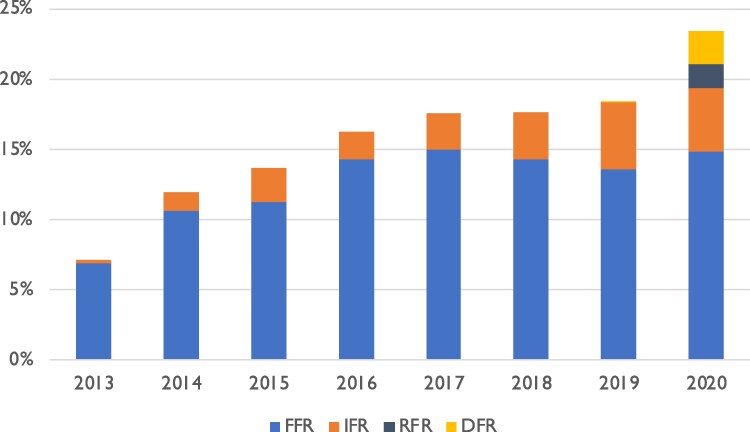
Annual use of flow assessment methods over time (FFR, fractional flow reserve; IFR, instantaneous wave-free ratio; RFR, resting full-cycle ratio; DFR, diastolic hyperaemia-free ratio)

Out of the 18 211 patients who underwent CABG in Sweden from 2013 to 2020, a total of 2869 (15.8%) had a functional assessment of stenosis with physiological flow measurements prior to surgery. Modalities used were FFR without further specification (*n* = 2353), instantaneous wave-free ratio (IFR, Philips, Amsterdam, The Netherlands) (*n* = 506), resting full-cycle ratio (RFR, Abbott Cardiovascular, Chicago, IL, USA) (*n* = 37), and diastolic hyperaemia-free ratio (Boston Scientific, Marlborough, MA, USA) (*n* = 52), with a majority (79.8%) being FFR (*Figure 1*). In 79/2869 (2.7%) of the flow assessment patients, two different methods were used. The proportion of patients with flow assessment increased from 7.2% in 2013 to 21.5% in 2020 (*Figure 2*). Baseline characteristics of patients with and without preoperative flow assessment are presented in *Table 1*. Patients with flow assessment were younger, less often female, more often had hypertension, diabetes, a history of PCI, and better LVEF. Flow assessment was more often conducted in patients with chronic coronary syndromes. In the flow assessment group, 54.9% of patients had an acute coronary syndrome less than 6 weeks before surgery, compared with 68.0% in the group without flow assessment. Median EuroSCORE II was lower in flow assessment patients [1.28 (0.89–1.99) vs 1.52 (1.01–2.52) %].

Median follow-up was 3.8 years (range 0–7.5). A total of 207 patients (1.1%) died within 30 days after CABG. During follow-up, 1605/18 211 patients died (8.8%), 592 (3.2%) had a post-discharge MI, 597 (3.3%) had a postoperative stroke, 2075 (11.5%) underwent new angiography, and 1095 (6.0%) underwent a new revascularization procedure. Of the new revascularization procedures, 95.5% were PCI.

### Perioperative variables

Patients with flow assessment had fewer peripheral anastomoses performed at surgery compared with patients without flow assessment (mean 3.0 ± 0.9 vs 3.2 ± 1.0, *P* < 0.001). Cardiopulmonary bypass was utilized in 98.8% of the patients, and did not differ between the intervention groups. However, CPB and aortic cross-clamp times were slightly shorter in the flow assessment group, CPB time 73 (IQR 57–93) vs 76 (IQR 60–96) min and aortic cross-clamp time 46 (IQR 36–60) vs 48 (IQR 38–62) min (both *P* < .001). There was similar utilization of any internal mammary artery in both groups, but more patients in the flow assessment group had bilateral internal mammary grafts (11.1% vs 4.7%, *P* < .001), and a higher number of patients had use of venous grafts in the no flow assessment group (*Table 2*).

### Outcome

#### 30-day mortality

Crude 30-day mortality per 100 patient-years was 1.0 in the flow assessment group and 1.2 in the no flow assessment group (*P* = .38). After multivariable adjustment, there was no significant difference in 30-day mortality risk between patients with flow assessment vs no flow assessment (aHR 1.41, 95% CI 0.93–2.14, *P* = .11).

#### Mid-term outcome

**Figure 3:**
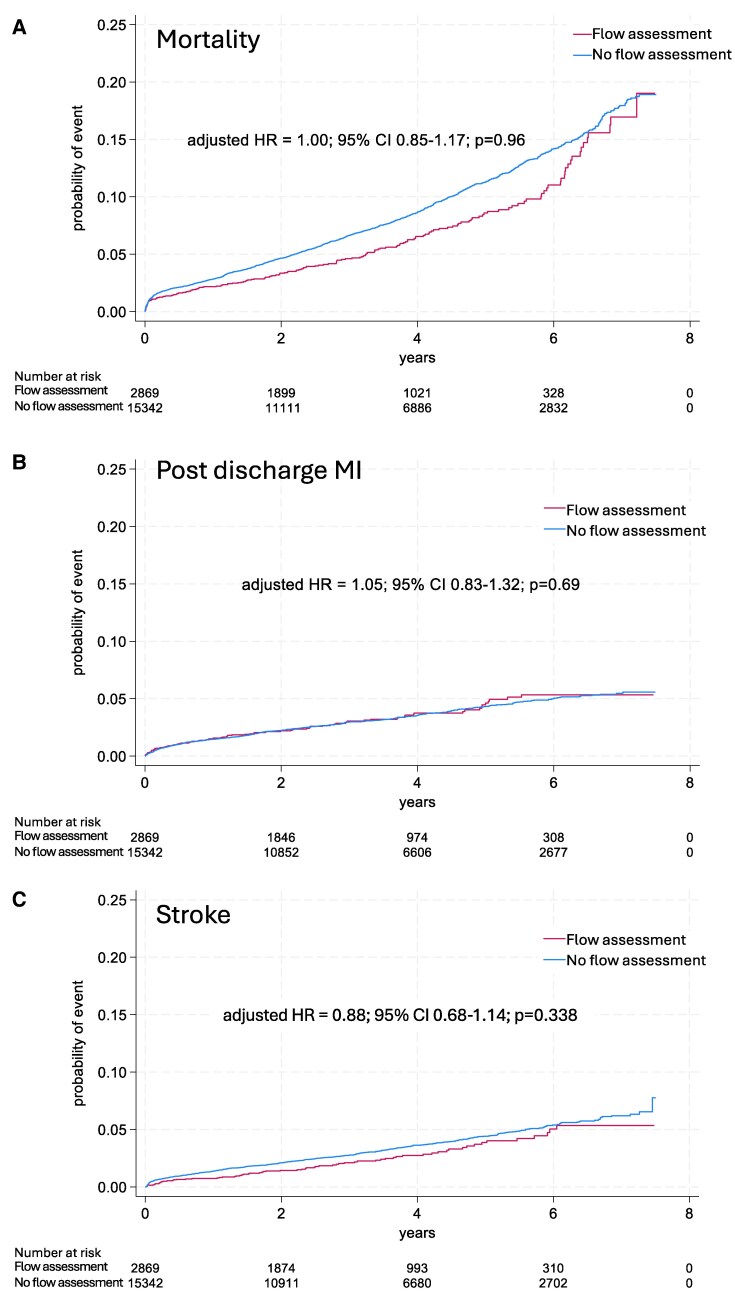
Unadjusted probability of mid-term outcomes presented in Kaplan–Meier curves, with adjusted hazard ratios from Cox regression analysis. (*A*) Post-discharge myocardial infarction (*B*), stroke (*C*) in coronary artery bypass grafting patients with and without preoperative flow assessment.

**Figure 4:**
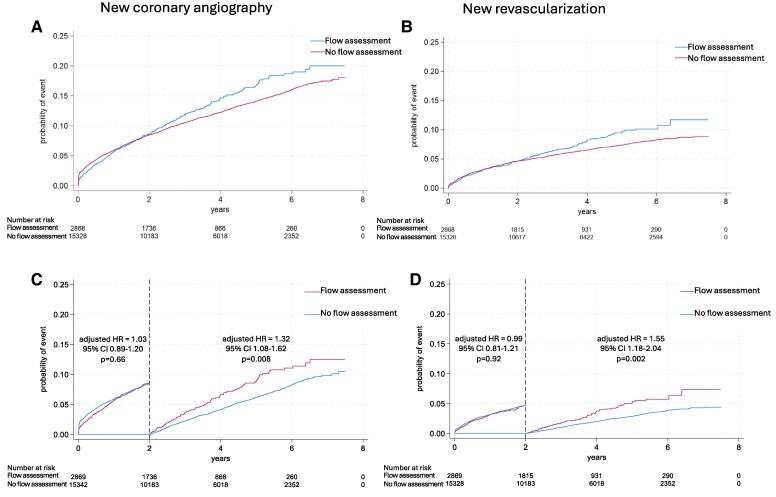
Unadjusted probability of (*A*) new coronary angiography and (*B*) new revascularization, in coronary artery bypass grafting patients with and without preoperative flow assessment during the entire follow-up. Unadjusted probability of (*C*) new coronary angiography and (*D*) new revascularization with landmark analysis. The curves are divided into 0–2 years and >2 years due to time-treatment interaction (*P* < .001). Adjusted hazard ratios from Cox regression analysis.

The crude incidence of all-cause death, post-discharge MI, and stroke were not significantly different between groups (*Table 3* and *Figure 3*). After multivariable adjustment, there was no significant difference in the risk for death, post-discharge MI, or stroke between patients with flow assessment vs no flow assessment. Adjusted HR for mortality in the flow assessment group vs no flow assessment was 1.00 (95% CI 0.85–1.17, *P* = 0.96), new MI aHR 1.05 (95% CI 0.83–1.32, *P* = .69), stroke aHR 0.88 (95% CI 0.68–1.14, *P* = .34). For new coronary angiography and new revascularization, there was no difference in adjusted risk during the first two postoperative years (aHR 1.03, 95% CI 0.89–1.20, *P* = .66 and aHR 0.99, 95% CI 0.81–1.21, *P* = .92, respectively). For the period after 2 years, flow assessment was associated with an increased risk for new coronary angiography (aHR 1.32, 95% CI 1.08–1.62, *P* = .008) (*Figure 4*). The *E*-value for repeat angiography after 2 years was 1.74. Flow assessment was also associated with new revascularization (aHR 1.55, 95% CI 1.18–2.04, *P* = .002), with an *E*-value of 2.06. The mediation analysis demonstrated that the number of anastomoses partially mediated the effect of flow assessment measurement on repeat revascularization, for details see Results section of the supplement.

**Table 3:** Incidence and hazard ratios for mid-term outcome variables in CABG patients with and without preoperative flow assessment with no flow assessment as reference

	Crude incidence^a^		Adjusted hazard ratio^b^ and 95% CI (flow assessment vs no flow assessment)	*P*-value
	Flow assessment (*n* = 2869)	No flow assessment (*n* = 15 342)		
Mortality	1.94	2.53	1.00 (0.85–1.17)	.96
Post-discharge MI	0.95	0.90	1.05 (0.83–1.32)	.69
Stroke	0.75	0.94	0.88 (0.68–1.14)	.34
New coronary angiography				
0–2 years	5.37	6.07	1.03 (0.89–1.20)	.66
>2 years	3.43	2.86	1.32 (1.08–1.62)	.008
New revascularization				
0–2 years	3.44	3.54	0.99 (0.81–1.21)	.92
>2 years	1.85	1.46	1.55 (1.18–2.04)	.002

^a^Per 100 patient-years.

^b^Adjusted for indication (acute coronary syndrome/chronic coronary artery disease), age, sex, body mass index, estimated glomerular filtration rate, previous percutaneous coronary intervention, left ventricular ejection fraction, priority (acute/non-acute), NYHA class, EuroSCORE II, previous myocardial infarction, diabetes, hypertension, heart failure, atrial fibrillation, previous stroke, chronic respiratory disease, renal failure, peripheral vascular disease, history of cancer, hyperlipidemia, and year of surgery.

MI, myocardial infarction; CI, confidence interval.

## Discussion

In summary, the use of flow assessment in the diagnostic work-up before CABG has increased in Sweden from 2013 to 2020. However, flow assessment before CABG was not associated with a clinical benefit but rather with a higher incidence of new angiography and new revascularization more than 2 years after surgery (*Structured Graphical Abstract*). Patients with flow assessment also received a lower number of distal anastomoses.

In PCI studies, revascularization based on visual estimation of lesion severity resulted in a higher number of lesions treated with stents and, importantly, a higher risk for MI due to increased risk for periprocedural complications and a low but persistent increased risk for stent thrombosis during follow-up, as compared with flow assessment-guided revascularization.^23^ The benefit of FFR guidance in PCI is arguably linked to a less-is-more concept of reduced stent burden when stenting only highly significant stenoses, which reduces the risk of stent-related complications during follow-up. Additionally, deferring PCI of functionally insignificant lesions has been reported to be safe, with low risk for adverse events.^4,24^

Undoubtedly, PCI and CABG are completely different revascularization techniques. In the present study in patients undergoing CABG, no flow assessment prior to surgery did not increase the risk for MI compared with flow assessment, despite the higher number of distal anastomoses. Furthermore, the safety of deferring grafting of visually significant but functionally insignificant lesions remains unclear. Possibly, in CABG, a more-is-more strategy may be advocated, parallel to the ‘surgical collateralization’ concept suggested by Doenst and Sigusch,^25^ noting that the benefit of CABG over PCI in chronic coronary syndromes may be attributed to prevention of new MIs occurring in vessels with previously non-flow limiting stenoses. Following this paradigm, the benefit of CABG over PCI in chronic coronary syndromes is not only revascularization but additionally protection against future MIs. Hence, Doenst and Sigusch suggest it would be of importance to supply as many coronary territories as possible with surgical collaterals in order to increase the extent of secured myocardium, assuming that the grafts remain patent.^25^

The main argument for flow assessment-guided CABG is that grafting of functionally significant lesions solely would reduce the risk for early or late graft failure by lowering the risk for competitive flow to the graft.^13^ One reason why flow assessment before surgery failed to show a positive effect in our study may be that we report a high proportion of vein graft use. Arterial grafts are considered more susceptible to competitive flow and would, therefore, benefit more from being grafted to a highly stenosed vessel than a venous graft, as discussed in detail in a state-of-the-art review by Spadaccio *et al*.^12^ In this material, radial arteries were used in less than 2% of cases, which is important as radial arteries may be even more affected by competitive flow than mammary arteries. Furthermore, bilateral mammary arteries were used in a minority of cases, and the fact that this does not translate into improved long-term outcomes should be interpreted with caution.

Accelerated progress of atherosclerosis in native coronary arteries, regardless of revascularization following CABG, has been described^26^ as increasing the risk for adverse events in untreated vessels. That may offset any potential benefit of fewer graft anastomoses. Thus, securing as many diseased vessels as possible at surgery may be beneficial. Our data support this reasoning as patients with flow assessment had fewer distal anastomoses and more new revascularization procedures late after surgery. The increased incidence of new angiography in patients with flow assessment indicates a higher incidence of re-angina. The lower number of distal anastomoses when using flow assessment was also described in a retrospective study by Toth *et al*.^7^, where fewer anastomoses were performed, especially to the right coronary territory.

Furthermore, the benefit of flow assessment-guided PCI was derived from patients with chronic coronary syndrome or assessment of non-culprit lesions in acute coronary syndrome^4-6^ whereas patients undergoing CABG often have acute coronary syndrome as the indication for surgery (66% of patients in our study). In patients with MI, recent PCI studies have shown that the use of flow assessment to guide PCI of non-culprit lesions did not improve outcomes and may be disadvantageous,^27,28^ as the haemodynamic significance of a non-culprit but vulnerable plaque may not be able to predict the risk for future MI. Therefore, it seems that previous data from patients with chronic coronary syndromes undergoing PCI cannot be extrapolated to a CABG population.

This study population includes only patients who underwent CABG. Patients with angiographic multivessel disease may be downgraded using flow assessment to single- or two-vessel disease. They are often not subject to a heart team discussion but continue to ad-hoc PCI, and these patients would not be identified in this study setup. Conversely, borderline visually significant stenoses may be confirmed to be physiologically significant, strengthening the indication for CABG. However, it is more common that flow assessment downgrades stenoses, as shown in the FAME 3 study, where 24% of stenoses were deemed functionally non-significant after FFR.^29^

### Limitations and strengths

One significant limitation of the current study is that we lack information on whether the available flow assessment measurements guided the surgeon, as this is not a variable available in the registry. The propensity for the individual surgeon to utilize preoperative flow assessment when deciding to graft or defer grafting likely varies with experience, attitudes, and department culture. Surgeons’ adoption of newer diagnostic tools in cardiac surgery is an interesting clinical question, but we have not been able to elucidate it with the current study setup.

Another limitation of the current study is that we lack high-resolution data on the FFR measurement results, so we cannot know how many vessels were assessed or the number of stenoses downgraded from angiographically significant to functionally non-significant. Potentially, there could be other reasons for not grafting a vessel, such as technical difficulties or lack of grafting material, which is not noted in the registry. However, the formal mediation analysis strengthens the association between flow assessment and new revascularization, mediated through fewer distal anastomoses.

Other limitations include the limited follow-up time and the significant imbalance between the number and the preoperative characteristics of patients who had flow assessment and those who did not, as well as the use of several methods to functionally assess the stenoses, although FFR was the dominant method. The limited follow-up time is mainly due to the relatively limited time since functional measurements were broadly implemented in Sweden, as shown in *Figure 2*. The choice of diagnostic method is not mandated by any national guidelines but at the discretion of the interventionist at the specific centre. The time period described in this study represents the implementation phase of these modalities, which may, to some extent, explain the imbalance between the studied groups. Unfortunately, the imbalance does confer potential residual confounding, which we have attempted to circumvent using multiple statistical methods.

This study is an observational retrospective analysis of registry data, and it is important to acknowledge its limitations. The most important ones are the intrinsic risk of coding errors, selection bias, and residual confounding from unmeasured confounders. However, the national healthcare registries are well-validated, and we used *E*-values to assess the risk of residual confounding. The *E*-value quantifies the minimum strength of association that an unmeasured confounder would need to have with both the exposure and the outcome to nullify the observed association. The *E*-value for repeat angiography after 2 years was 1.74. This implies that any unmeasured confounding variable would need to be associated with both undergoing repeat angiography and flow assessment, at least 1.74 times stronger than the associations we observed, to negate our findings. Similarly, the *E*-value for repeat revascularization was 2.06, indicating that an unmeasured confounder, or the multiplied effect of several unmeasured confounders, must be at least 2.06 times stronger associated with both undergoing revascularization and flow assessment to change the risk estimate to the null. These results make our findings moderately robust against residual confounding.^21,30^ Furthermore, as stated by Toth *et al*.,^7^ a randomized controlled study with enough statistical power to adequately answer whether angiography or FFR is superior in guiding CABG would require over 7000 patients, making it highly unlikely to occur.

However, there are also several strengths, as this analysis represents a real-world nationwide experience over a long time, yielding a large number of patients with minimal loss of follow-up. Data are retrieved from well-validated mandatory nationwide registries with excellent coverage. However, the SWEDEHEART registry has been developed over time, starting with a limited number of variables, which have increased. This explains why values regarding operation times and grafting details are missing in *Table 2*. Unlike previous observational studies, we have estimated the amount of residual confounding necessary to annul our risk estimates using *E*-values.

## Conclusions

This study suggests that there is no significant clinical benefit with preoperative flow assessment using currently recommended cut-off levels in CABG—unlike the well-established benefit of FFR guidance in PCI. The results question whether flow assessment with currently used cut-offs is applicable in CABG, and warrants further prospective studies.

## Supplementary Material

ehaf327_Supplementary_Data

## Declarations

### Data Availability

Data is owned by a third party. The data underlying this article were provided by SWEDEHEART and national healthcare registries in Sweden by permission. Data will be shared on request to the corresponding author with permission of SWEDEHEART and the Swedish National Board of Health and Welfare.
