# Supplementary material for: Coronary artery bypass grafting with or without preoperative physiological stenosis assessment: a SWEDEHEART study
Source: Eur Heart J. 2025 May 16;46(32):3214–23. doi: 10.1093/eurheartj/ehaf327 (PMC12369819; doi:10.1093/eurheartj/ehaf327)
Supplement: ehaf327_Supplementary_Data [file ehaf327_supplementary_data.docx]

Coronary artery bypass grafting with or without preoperative physiological stenosis assessment: a SWEDEHEART study

*Emma C Hansson, Elmir Omerovic, Dimitrios Venetsanos, Joakim Alfredsson, Andreas Martinsson, Björn Redfors, Amar Taha, Susanne J Nielsen, Anders Jeppsson*

# Supplement

## Statistical methods

We performed a causal mediation analysis to assess whether the relationship between flow assessment (exposure) and repeat revascularization (outcome) is mediated by the number of anastomoses (mediator). The analysis was conducted using the mediation package in R (1). This package allows for the estimation of the Average Causal Mediation Effect (ACME), which quantifies the indirect effect of the treatment through the mediator, as well as the Average Direct Effect (ADE), which represents the direct effect of the treatment on the outcome independent of the mediator. For this analysis, we fit two models:

A mediator model, where the number of anastomoses was regressed on flow assessment and relevant covariates.

An outcome model where repeat revascularization was regressed on flow assessment, the number of anastomoses, and the same set of covariates used in the mediator model.

Both models were adjusted for potential confounders, including age, sex, comorbidities, and other clinical factors relevant to the patient population. The mediation analysis used non-parametric bootstrapping with 1000 simulations to estimate the 95% confidence intervals for the ACME, ADE, and total effect.

Since the outcome was modeled using Cox proportional hazards regression, we extracted the linear predictor from the Cox model to fit a linear regression model for the mediation analysis. The linear predictor, representing the hazard ratios logarithm, reflects the covariates influence on the risk of repeat revascularization. The linear predictor allows the mediation analysis to appropriately account for the effect of flow assessment on revascularization while adjusting for the mediator.

We further quantified the proportion of the total effect mediated by the number of anastomoses to determine how much flow assessments effect on revascularization this mediator explains. The mediation analysis results are reported with corresponding confidence intervals and p-values.

Additionally, to formalize the causal structure of the relationships between the variables, we used Directed Acyclic Graphs (DAGs), created using the dagitty package in R (2). DAGs helped to clarify the causal assumptions and identify any confounding variables that should be included in the analysis.

## Results

### Mediation Analysis

The mediation analysis demonstrated that the number of anastomoses partially mediates the effect of flow assessment measurement on repeat revascularization. The Average Causal Mediation Effect (ACME), representing the indirect effect of flow assessment on revascularization through the number of anastomoses, was estimated at -4.9% (95% CI: -5.8%, -4.0%, p < 0.001). This indicates that flow assessment measurement significantly reduces the number of anastomoses, increasing the likelihood of repeat revascularization.

The Average Direct Effect (ADE), representing the direct effect of flow assessment on revascularization independent of the mediator, was estimated at -3.0% (95% CI: -3.3%, -3.0%, p < 0.001). This suggests that, even after accounting for the number of anastomoses, flow assessment significantly increases the likelihood of repeat revascularization.

The Total Effect, which combines the direct and indirect effects of flow assessment on revascularization, was estimated at -8.2% (95% CI: -9.1%, -7.0%, p < 0.001). This demonstrates that flow assessment significantly increases the overall likelihood of repeat revascularization.

The proportion of the total effect mediated by the number of anastomoses was estimated at 59.5% (95% CI: 54.7%, 64.0%, p < 0.001), indicating that over half of the effect of flow assessment on revascularization is mediated through its influence on the number of anastomoses.

## Supplemental table 1

ICD codes used for baseline comorbidities and outcomes

|  | ICD 9 | ICD 10 |
| --- | --- | --- |
| Baseline comorbidities | | |
| Diabetes | 250 | E10, E11 |
| Hypertension | 401, 402, 403, 404, 405 | I10, I11, I12, I13, I14, I15 |
| Heart failure | 425, 428 | I110, I130, I132, I255 |
| Atrial fibrillation | 427D | I48 |
| Previous stroke | 431, 434, 436, 432X | I61, I62, I63, I64, I69 |
| History of cancer | 140-208 | C00-C97 |
| Chronic respiratory disease | 490, 491, 492, 493, 494, 495, 496 | J40, J41, J42, J43, J44, J45, J46, J47 |
| *Outcome* | | |
| *MI* | *410* | *I210, I211, I212, I213, I214, I219* |
| *Stroke* | 431, 434, 436, 432X | I61, I62, I63, I64, I69 |

## Supplemental figure legend

### Figure S1

Directed acyclic graphs showing the relationship between variables in the formal mediation analysis.

## Supplemental figure

### Figure S1


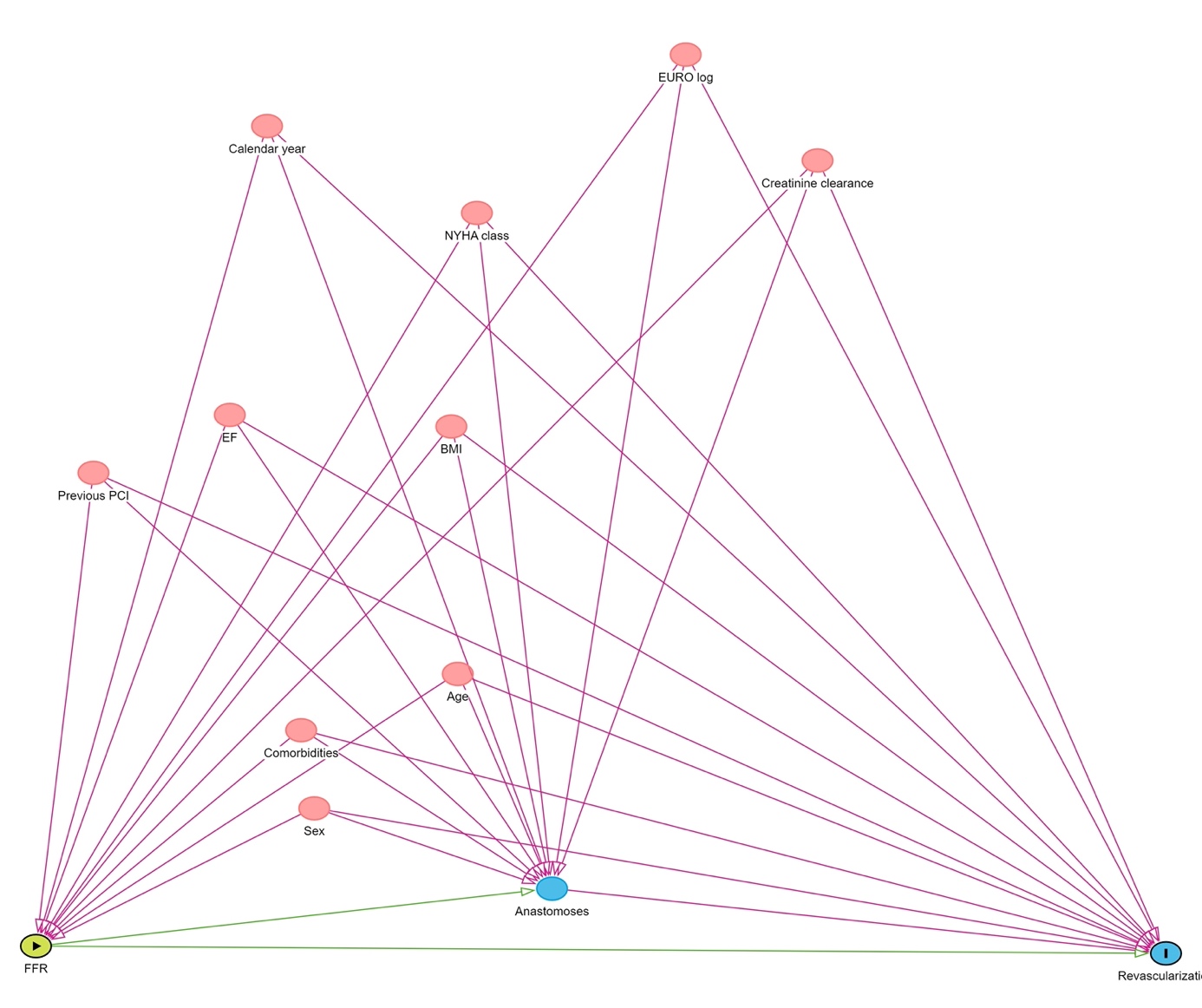


## References

1. Tingley D, Yamamoto T, Hirose K, Keele L, Imai K. mediation: R Package for Causal Mediation Analysis. Journal of Statistical Software. 2014;59(5):1 - 38.

2. Textor J, van der Zander B, Gilthorpe MS, Liskiewicz M, Ellison GT. Robust causal inference using directed acyclic graphs: the R package dagitty. Int J Epidemiol. 2016;45(6):1887-94.
